# Natural bone-mimicking nanopore-incorporated hydroxyapatite scaffolds for enhanced bone tissue regeneration

**DOI:** 10.1186/s40824-022-00253-x

**Published:** 2022-02-25

**Authors:** Chansong Kim, Jin Woong Lee, Jun Hyuk Heo, Cheolhyun Park, Dai-Hwan Kim, Gyu Sung Yi, Ho Chang Kang, Hyun Suk Jung, Hyunjung Shin, Jung Heon Lee

**Affiliations:** 1 School of Advanced Materials Science and Engineering, Sungkyunkwan University (SKKU), Suwon, 16419 Republic of Korea; 2 Research Center for Advanced Materials Technology, Sungkyunkwan University (SKKU), Suwon, 16419 Republic of Korea; 3 Probiomimetic Research Institute, Bundang Technopark, Seongnam, 13219 Republic of Korea; 4 Department of Energy Science, Sungkyunkwan University (SKKU), Suwon, 16419 Republic of Korea; 5 Biomedical Institute for Convergence at Sungkyunkwan University, Sungkyunkwan University (SKKU), Suwon, 16419 Republic of Korea; 6 Institute of Quantum Biophysics (IQB), Sungkyunkwan University (SKKU), Suwon, 16419 Republic of Korea

**Keywords:** Natural bone-mimicking, Nanopore, Hydroxyapatite, Scaffold, Bone graft material

## Abstract

**Background:**

A considerable number of studies has been carried out to develop alloplastic bone graft materials such as hydroxyapatite (HAP) that mimic the hierarchical structure of natural bones with multiple levels of pores: macro-, micro-, and nanopores. Although nanopores are known to play many essential roles in natural bones, only a few studies have focused on HAPs containing them; none of those studies investigated the functions of nanopores in biological systems.

**Method:**

We developed a simple yet powerful method to introduce nanopores into alloplastic HAP bone graft materials in large quantities by simply pressing HAP nanoparticles and sintering them at a low temperature.

**Results:**

The size of nanopores in HAP scaffolds can be controlled between 16.5 and 30.2 nm by changing the sintering temperature. When nanopores with a size of ~ 30.2 nm, similar to that of nanopores in natural bones, are introduced into HAP scaffolds, the mechanical strength and cell proliferation and differentiation rates are significantly increased. The developed HAP scaffolds containing nanopores (SNPs) are biocompatible, with negligible erythema and inflammatory reactions. In addition, they enhance the bone regeneration when are implanted into a rabbit model. Furthermore, the bone regeneration efficiency of the HAP-based SNP is better than that of a commercially available bone graft material.

**Conclusion:**

Nanopores of HAP scaffolds are very important for improving the bone regeneration efficiency and may be one of the key factors to consider in designing highly efficient next-generation alloplastic bone graft materials.

**Supplementary Information:**

The online version contains supplementary material available at 10.1186/s40824-022-00253-x.

## Background

Bone graft materials (BGMs) can regenerate fresh bones around defects caused by bone infections, surgery, trauma, and congenital malformations [1, 2]. The BGMs used for bone therapies are generally classified into autografts, allografts, xenografts, and alloplastic materials, but these substitutes are far from ideal because of their intrinsic structure and limitations. For example, because autografts are natural bones extracted from the same individual receiving the graft, they exhibit the lowest risk originating from immune reaction or infection [3]. However, the use of autografts requires additional surgeries to extract them, which can cause additional pain and expenditure [4, 5]. Allografts and xenografts are natural bones obtained from individuals of the same species and nonhuman species, respectively. However, they pose risks of disease transmission and occurrence of immune response [6]. Alloplastic materials are artificially synthetized BGMs, which have been actively used because they are cost-effective and have reduced chances of immune reactions [7, 8].

Hydroxyapatite (HAP) is one of the most commonly used alloplastic BGMs. It is the major inorganic component of natural bones and is most frequently utilized [1, 9–15] because it not only exhibits excellent bioactivity with osteoconduction and osteoinduction in biological systems but also releases essential ions (e.g., calcium and phosphate ions), resulting in stimulation of cell growth and differentiation [14, 16–19]. Owing to its outstanding biocompatibility, HAP can interact with natural tissues without causing significant inflammatory reactions [20–22]. In addition, certain characteristics of HAP, such as the size, morphology, and crystallinity, can be easily controlled under mild conditions [23–27]. However, synthesized HAPs are structurally different from natural bones, which may have negative effects, such as increasing ectopic bone formation [28–30]. Thus, even if the synthesized HAP can mimic the dimensions and components of natural bone, it is very important to imitate the structural properties of natural bone.

Most natural materials, including bone, wood, and shell, have hierarchical architectures with multiple levels of pores: macro-, micro-, and nanopores [31–33]. The multi-level porosity of natural bone is important for effective nutrient delivery; cell migration, proliferation, and differentiation; and vascularization in biological systems [34]. Macro- and micro-porous structures of natural bones facilitate the osteogenic differentiation for bone regeneration because these pores can assist the spreading and elongation of stem cells [35, 36]. The nanoporous structures of bones mainly provide a large surface area, which is advantageous for protein adsorption, including that of growth factors, such as bone morphogenetic protein 2 and vascular endothelial growth factor, for effective bone regeneration [37–39]. They can also change the morphology of macrophages by providing different immune environments. Moreover, they can induce the recruitment and differentiation of osteoblasts in the early stages of bone formation [40].

Several researchers primarily investigated the design of the internal porous structures of materials, including HAP. For example, the salt-templated method has been used to fabricate macropore-embedded scaffolds. This material effectively recruits host immune cells [41]. In addition, a microporous structure was fabricated using polycaprolactone for bone tissue engineering [14, 42, 43]. However, it is very challenging to introduce nanopores into materials using template-assisted strategies because they cause significant coalescence and destruction of pores [44]. Hence, in contrast to the relatively abundant studies on scaffolds with macro- and micro-pores, only a few studies related to the fabrication of nanoporous scaffolds and their biological activities have been reported [45–47]. Furthermore, the macro- and micro-scale pores in HAP scaffolds improve cell attachment and bio-mineralization [48–50]. However, the biological effects of nanopores with sizes in the tens of nanometers range, similar to those in natural bones in HAP scaffolds, have rarely been studied [51–57].

**Fig. 1:**
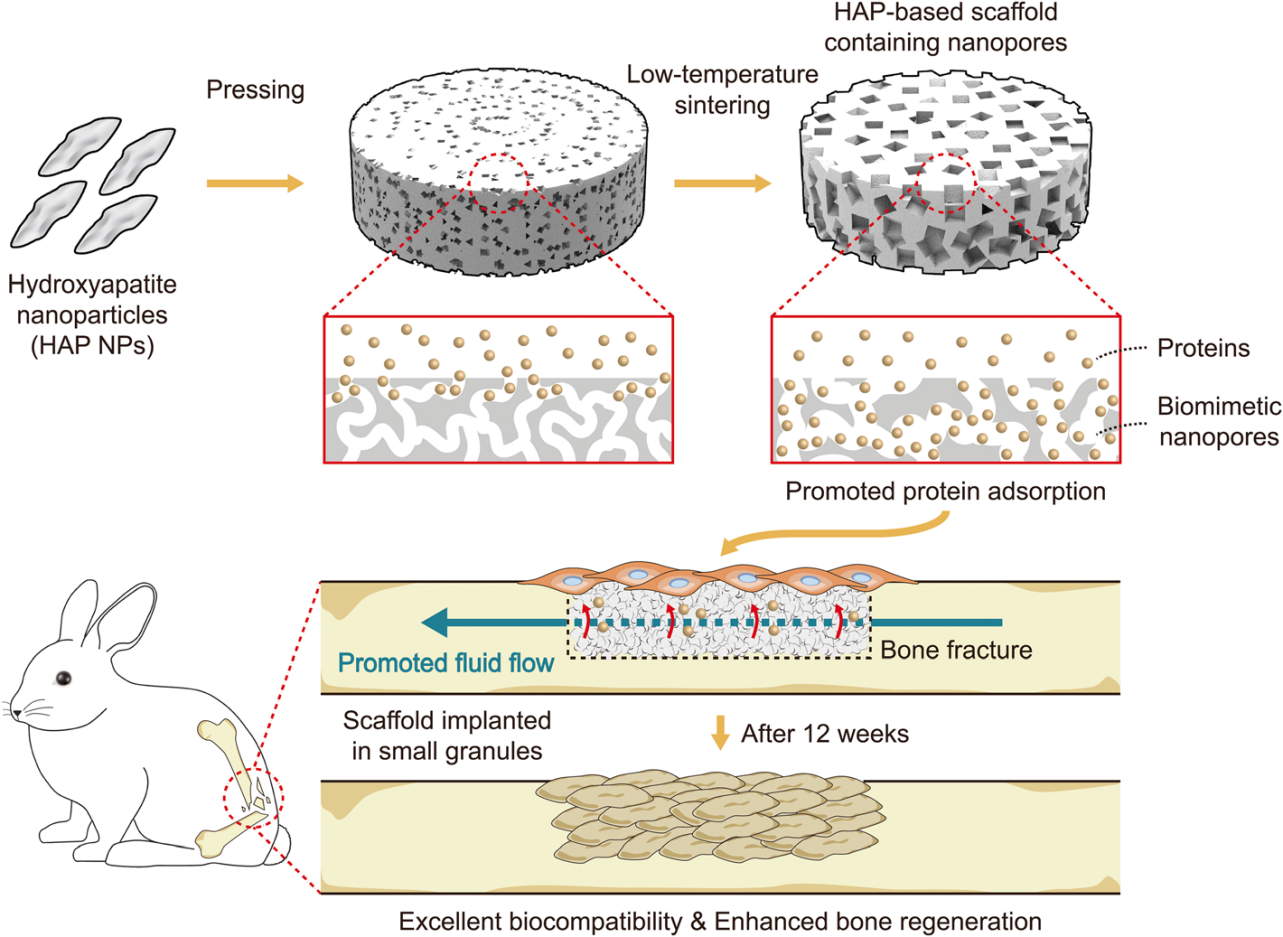
Schematic representation of the method used for fabricating SNPs that promote bone regeneration. The size of the nanopores prepared through this method (~ 16.5–30.2 nm) is comparable to that of nanopores in natural bones

In this study, we propose a simple method to fabricate HAP-based scaffolds containing nanopores (SNPs) and evaluate the effects of the nanopores on the bone regeneration. Nanopores could be introduced into the HAP-based scaffold by simply pressing HAP nanoparticles and sintering within a low-temperature range (Fig. 1). Generally, ceramic particles are sintered above 1000 °C, at which temperature the nanopores present between the HAP nanoparticles in contact are removed [58, 59]. We hypothesized that a low-temperature sintering could be applied to fabricate nanopores in HAP-based scaffolds. To test our hypothesis, we controlled the sintering temperature from 100 to 500 °C and found that nanopores with sizes between 16.5 and 30.2 nm could be successfully introduced into the HAP-based scaffold. Among them the fabricated nanopores with the size of ~ 30.2 nm were similar to those in natural bones [60, 61]. The nanopores of HAP-based scaffolds helped enhance both proliferation and differentiation rates of preosteoblasts. Moreover, by investigating the water and protein adsorption efficiencies of SNPs, we showed important roles of the nanoporous structures in bone regeneration. Finally, we demonstrated that SNPs can be a novel bone-generating substitute for damaged bone and can promote regeneration in biological systems using small animals.

## Methods

### Materials

Sodium phosphate monobasic dihydrate (NaH_2_PO_4_·2H_2_O) (99.0%), sodium hydroxide (NaOH) (98%), and dimethyl sulfoxide (99.0%) were purchased from Samchun (Republic of Korea). Calcium nitrate tetrahydrate (Ca (NO_3_)_2_·4H_2_O) (98.0%), urea (99.0%), and phosphoric acid (H_3_PO_4_) (85.0%) were purchased from Junsei (Japan). Calcium hydroxide (Ca (OH)_2_) (98.0%) was purchased from Acros Organics (USA). Deionized (DI) water (18.2 MΩ cm) was prepared using a Sartorius Arium®Pro Ultrapure water system and was used for all experiments. All reagents were used without further purification. All glassware was cleaned using aqua regia before use. As necessary, special care was employed in handling aqua regia. Mouse MC3T3-E1 preosteoblasts were used in this study were purchased from American Type Culture Collection (USA). Alpha minimum essential medium (α-MEM; Gibco, USA) was used to grow the cells. The 3-(4,5-dimethylthiazol-2-yl)-2,5-diphenyltetrazolium bromide (MTT) and alkaline phosphate (ALP) assay kits used to evaluate the proliferation and ALP activity of the cells were purchased from Sigma-Aldrich (USA). All materials were used without further purification.

### Synthesis of HAP

HAP was synthesized using a slightly modified method [12]. For the synthesis, 9.63 g of Ca (NO_3_)_2_·4H_2_O, 5.96 g of NaH_2_PO_4_·2H_2_O, and 4.856 g of urea were dissolved in 2 L of DI water. The pH of the reaction solution was adjusted in the range of 10–11 using a 2 M NaOH stock solution. The transparent solution turned white and opaque. After thorough mixing, the mixture was incubated at 90 °C for 36 h for further reaction. The precipitated HAP was washed three times with DI water. Finally, the washed HAP was lyophilized overnight to obtain the NPs in a dried powder form.

### Fabrication of SNPs using low-temperature sintering

SNPs were fabricated by pressing and sintering at different temperatures. Approximately 0.125 g of HAP was transferred into a mold with a diameter of 1 cm and pressed with a pressure of 350 kgf for 3 min. The pressed HAP pellet was sintered for 6 h at a certain temperature with an increment of 5.33 °C/min. Non-sintered pressed HAP pellets were not suitable for liquid-based experiments because they could not maintain their original structure and crumbled owing to the lack of bonding between the HAP particles. As HAP was already treated with a heating process at approximately 100 °C during the synthesis step, sintering at 100 °C hardly affected its original characteristics. Therefore, to minimize the bonding between the HAP particles for liquid-based experiments, the HAP pellet sintered at 100 °C was used as a control in this study.

### Protein and water adsorption test

Pierce bicinchoninic acid (BCA) assay kit (Thermo Fisher Scientific, USA) was used to determine the protein adsorption efficacy of the SNP. To allow the SNPs to adsorb proteins in the cell culture media, samples were immersed in α-MEM containing 10% fetal bovine serum (FBS) and 1% antibiotics (penicillin/streptomycin). After incubation at 37 °C, the SNPs were gently washed twice with phosphate-buffered saline (PBS). The adsorbed proteins were lysed with a 0.1% Triton X-100 solution. Approximately 25 μL of the lysate was mixed with 200 μL of the supplied BCA working solution. This mixture was incubated at 37 °C for 30 min. Finally, the absorbance at 562 nm (A562 nm) was measured using a microplate.

### Fixation, immunostaining, and confocal imaging

An actin cytoskeleton/focal adhesion staining kit (FAK1000, EMD Millipore, USA) was used to immunostain MC3T3-E1 cells cultured on the SNPs [62–65]. After incubating 30,000 cells on SNPs located in a 24-well plate for 8 h at 37 °C, the SNPs were transferred to a new plate and washed twice with PBS. The cells were subsequently fixed with a fixative solution (4% paraformaldehyde in PBS) at 27 °C for 20 min, washed twice with a wash buffer (0.05% Tween 20 in PBS), and permeabilized with 0.1% Triton X-100 in PBS for 5 min. After washing twice with the wash buffer, the samples were treated with a blocking solution (1% bovine serum albumin in PBS) for 30 min. Subsequently, the blocking solution was replaced with 3 μL of the supplied vinculin monoclonal antibody and 297 μL of the blocking solution and incubated for 3 h, followed by rinsing three times with the wash buffer for 5 min (each cycle). 30 μL of the secondary antibody (goat anti-mouse IgG (H + L) fluorescein isothiocyanate conjugated, EMD Millipore) and 7.5 μL of the supplied tetramethylrhodamine (TRITC)-conjugated phalloidin (0.06 μg/μL) in 262.5 μL of PBS were then added to the SNPs and incubated for 1 h, followed by washing three times with the wash buffer for 5 min (each cycle). Subsequently, 3 μL of the supplied 4′,6-diamidino-2-phenylindole in 297 μL of PBS was added to the samples, which were then incubated for 5 min. Finally, the samples were transferred to a coverglass-bottom Petri dish. A confocal laser microscope (TCS SP8, Leica, Germany) was used to image the actin filaments and focal adhesions [63].

### Mechanical properties

Compression tests were performed on the prepared SNPs using an Instron 3369 universal testing machine (Norwood, MA, USA). The compressive strength tests were performed at a crosshead speed of 1 mm/min using cylindrical samples (diameter: 10 mm; height: 60 mm). Five samples were tested for each SNP, and then the average of the obtained values was calculated.

### Evaluation of cell proliferation and differentiation rates

MTT and ALP assays were utilized to evaluate the cell proliferation and differentiation rates of preosteoblasts cultured on SNPs. Each SNP was placed in a 24-well plate and immersed in α-MEM containing 10% FBS and 1% antibiotics (penicillin/streptomycin), followed by incubation at 37 °C for 24 h to allow the SNPs to fully adsorb proteins before the MC3T3-E1 cells were spread on the samples. After incubating 30,000 cells on the SNPs, each sample was transferred into a new well plate and washed twice with PBS. To evaluate cell proliferation, a 0.5-mg/mL MTT solution and dimethyl sulfoxide were applied successively for 3 and 1 h, respectively, and then the absorbance at 570 nm (A570 nm) was measured using a microplate. Additionally, to evaluate ALP activity, cells were lysed with a 0.1% Triton X-100 solution for 1 h. Then, 30 μL of the lysate was mixed with 140 μL of an ALP reaction buffer and 30 μL of a 4-mg/mL *p*-nitrophenyl phosphate solution. Finally, the absorbance at 405 nm (A405 nm) was measured using a plate reader. During the culturing of MC3T3-E1 cells, the medium was exchanged with a fresh medium every 2 days.

### Biocompatibility and bone regeneration efficiency

For the biocompatibility and implantation tests, female albino rabbits (mass: 2–3 kg) were purchased from Dae Han Bio Link Co., Ltd. (Republic of Korea). Animal testing was carried out in accordance with the legal guidelines at the Dental Material Testing Development Center at Kyung Hee University with the approval of the Ministry of Food and Drug Safety of Korea.

An intracutaneous injection test was conducted according to the International Organization for Standardization (ISO) 10,993–10 test guidelines to determine the biocompatibility of SNP500 (SNP sintered at 500 °C). Furthermore, 4 g/20 mL of the SNP was incubated in a 0.9% sodium chloride solution (saline) and cotton seed oil (CSO) as polar and nonpolar solvents, respectively. The extracts were eluted at 120 °C for 1 h. Thereafter, 200 μL of each extract was injected into the back of a mouse and the erythema and edema reactions were observed.

A local lymph node assay was conducted according to defined test specifications (OECD 442 B; ISO 10993-10) to determine the biocompatibility of SNP500 based on skin sensitization. The SNP was incubated in saline and CSO at a concentration of 4 g/20 mL. The extracts were eluted at 120 °C for 1 h. After applying 25 μL of each extract to the earlobe of the mouse, the thickness and mass of the pinna were measured. Subsequently, the auricular lymph nodes were crushed in 15 mL of PBS to separate the lymphocytes, and 100 μL was transferred to a 96-well plate. After centrifuging the well plate at 300 RCF for 10 min, the supernatant was removed and the samples were completely dried at 60 °C. Subsequently, a FixDenat solution and anti-BrdU-POD working solution were added to the lymphocytes and incubated at 25 °C for 30 and 90 min, respectively. After washing three times with 200 μL of a PBS solution, 100 μL of the substrate solution was added for 30 min, and then the absorbance at 370 nm (A370 nm) was measured using a plate reader. The stimulation index (SI) was calculated using the following formula:

SI = A370 nm_experimental_/A370 nm_NC_.

To compare the degrees of immune reactions, a 3% 1-choro-2,4-dinitrochlorobenzene (DNCB)–acetone olive oil (AOO) solution, which induces an allergic reaction, was used as a positive control. Saline, CSO, and AOO were used as negative controls.

To evaluate the tissue response to implantation and bone regeneration efficiency, SNP500 was implanted in 4 albino rabbits according to the ISO 10993-6 guidelines. In this test, commercial bone graft material (cBGM) (0701 M + G01, Biomatlante, France), which is one of the most commonly used synthetic bone graft products, was used as a control sample. After carefully generating puncture sites on the rabbit tibia, cBGM and SNP were sufficiently filled in the perforated sites through polypropylene tubes (inner diameter: 2.90 mm; length: 4 mm). After 12 weeks, the average length of the newly formed bone and tissue responses around the implanted samples were observed.

### Characterizations

Field-emission scanning electron microscopy (SEM; JSM-7600F, JEOL, Japan) and transmission electron microscopy (TEM; JEM-2100F, JEOL) were used to determine the surface morphology of HAP [66]. Field-emission SEM was conducted at the MEMS·Sensor Platform Center of Sungkyunkwan University. A dynamic light scattering analysis (ZetaSizer Nano ZS90, Malvern Panalytical Ltd., UK) was conducted to evaluate the hydrodynamic size. X-ray diffraction (XRD; D8 Advance, Bruker, USA) and Fourier-transform infrared (FTIR; IFS-66/S, Bruker) spectroscopy analyses were conducted to characterize the fabricated SNPs. Mercury intrusion porosimetry (MIP; ASAP 2460, Micromeritics, USA) was performed to measure the size and total area of the pores embedded in the SNPs. For the characterization, each sample was dried overnight at 90 °C under vacuum before the measurements.

### Statistical analysis

Obtained results are presented as the mean value ± standard deviation. Statistical analysis was conducted with Microsoft Excel software using an unpaired t-test. *p* < 0.05 was considered statistically significant.

## Results

### Characterization of the synthesized HAP

**Fig. 2:**
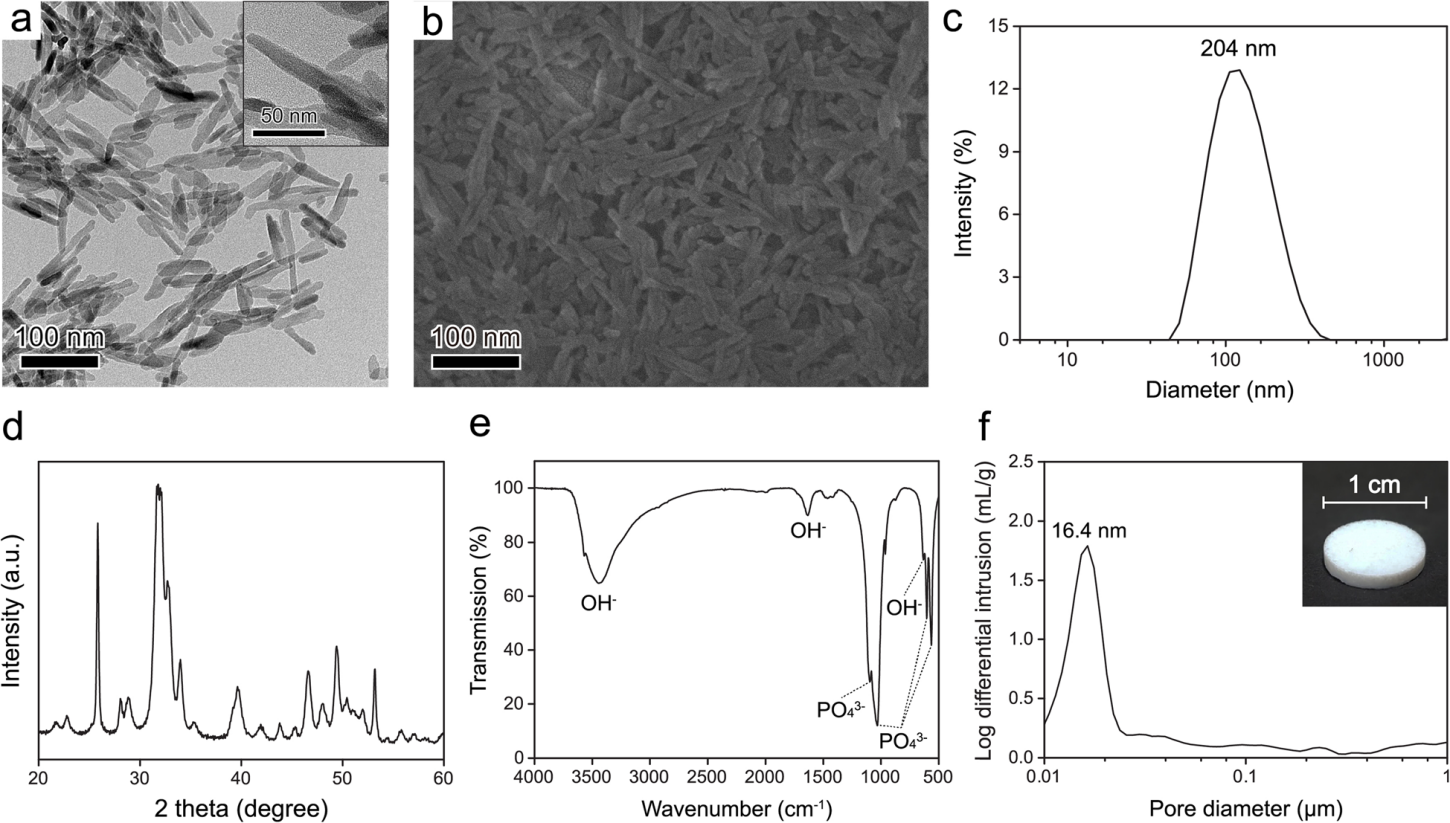
**(a)** TEM (inset: enlarged image) and (**b**) SEM images of the synthesized HAP. **(c)** Hydrodynamic size, (**d**) XRD pattern, and (**e**) FTIR absorption pattern of HAP. **(f)** Pore size distribution of HAP after pressing (inset: image of the pressed HAP)

HAP NPs were synthesized to fabricate the SNPs (Fig. 2). The HAP NPs exhibited elongated needle-like structures with an average hydrodynamic size of 204 nm (Fig. 2a–c). The XRD pattern had major peaks at 25.8°, 31.7°, 32.9°, 34.1°, 39.8°, 46.7°, 49.5°, and 53.1°, which suggested that the synthesized NPs were composed of HAP. The XRD pattern did not indicate other phases (Fig. 2d) [12, 67]. The FTIR spectrum exhibited transmission peaks at 3570, 3420, 1650, and 630 cm^− 1^ and at 1094, 1040, 960, 603, and 565 cm^− 1^, which correspond to the OH^−^ and PO_4_^3−^ groups of HAP, respectively (Fig. 2e) [68, 69]. A cylindrical pellet with a diameter of 1 cm was prepared using a pressing process (see the experimental section for detailed information). MIP measurements showed nanopores with a size of 16.4 nm formed in the prepared pellet (Fig. 2f).

### Biomimetic fabrication of the nanoporous structure in natural bone

**Fig. 3:**
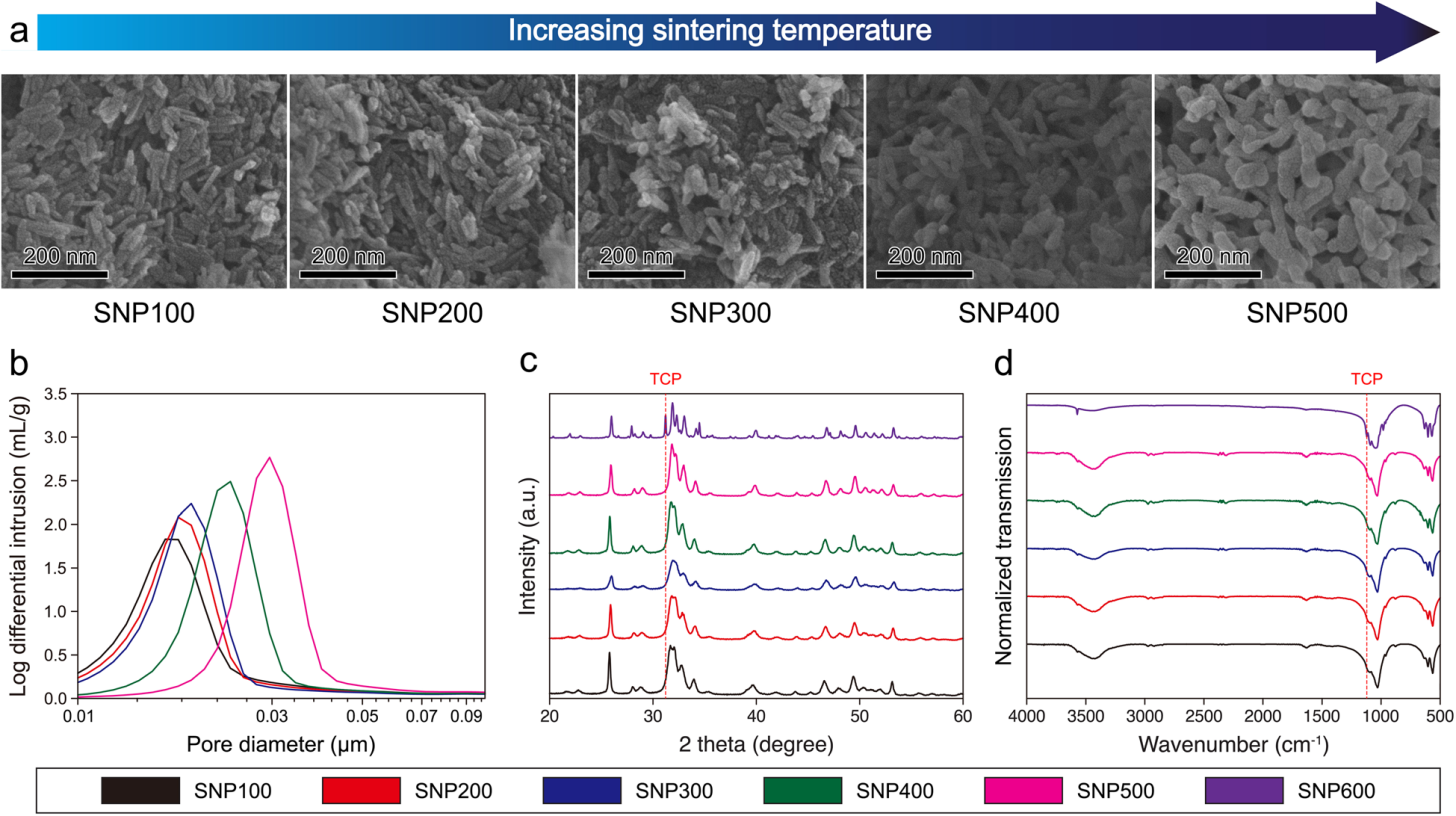
(**a**) SEM images, (**b**) internal pore size distributions, (**c**) XRD patterns, and (**d**) FTIR transmission patterns of SNPs sintered at different temperatures

To bind the HAP NPs together and control the size of the pores embedded in the SNPs, we sintered the pellets at different temperatures. Figure 3 shows the physicochemical characteristics of the SNPs sintered at different temperatures. The SEM analysis showed that the morphology of HAP became blunt and that the grain size increased with the sintering temperature (Fig. 3a). Notably, both the size and total area of SNP pores could be finely tuned from 16.5 to 30.2 nm and 126.6 to 75.2 m^2^/g, respectively, with a constant porosity (Fig. 3b and S1), likely because, once the SNP was sintered, the nanopores agglomerated, leading to a decrease in the surface area. In particular, the average size of the pores in the SNP sintered at 500 °C was approximately 30.2 nm, which is very similar to that of the pores in natural bones [70]. The compressive strength of the SNP with a pore size of 30.2 nm was highest compared to the other pellets (Fig. S2). However, both XRD and FTIR spectroscopy analyses indicated a phase transition from HAP to tricalcium phosphate (TCP) beyond the sintering temperature of 500 °C; a new peak appeared at 31.1° in the diffraction pattern and 1120 cm^− 1^ in the IR transmission spectrum (Fig. 3c and d) [67–69, 71]. As our study focused on the investigation of the effect of natural bone-mimicking nanopores in the HAP scaffold rather than the influence of the phase transition from HAP to TCP, SNPs sintered above 500 °C were not analyzed.

### Evaluation of the cell proliferation and differentiation rates

**Fig. 4:**
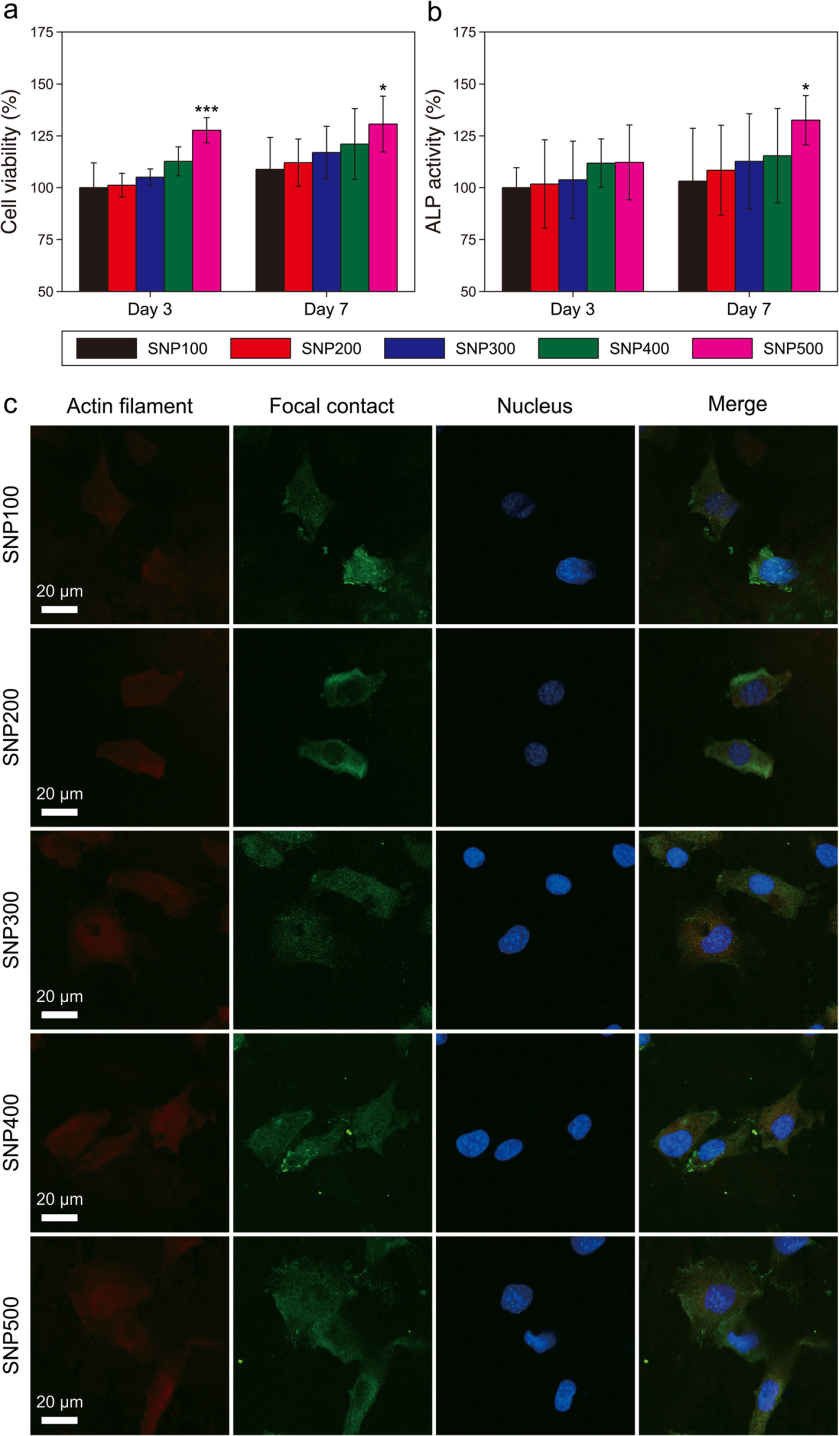
**(a)** Cell viability and (**b)** ALP activity of MC3T3-E1 cells cultured on SNPs prepared at different sintering temperatures. The data are presented as ratios (%) with respect to the SNP100 values. The statistical significance was derived relative to the results of SNP100 (*n* = 3; *P < 0.05; ***P < 0.001). **(c)** Confocal images of immunofluorescence-stained actin filaments, focal contacts, and nuclei in MC3T3-E1 cells cultured on SNPs

To determine the effect of the SNP pore size on the bone regeneration, we cultured mouse MC3T3-E1 preosteoblasts on the surface of SNPs prepared at different sintering temperatures. The proliferation and differentiation rates of the cells cultured on each SNP were measured using MTT and ALP assays for 3 and 7 days, respectively [72–74]. Both cell viability and ALP activity on the SNPs increased with the sintering temperature (Fig. 4a and b). These results suggest that SNP500, with nanopores similar to those of natural bones, is most suitable for the promotion of both proliferation and differentiation of preosteoblasts [75–77]. Furthermore, we determined the morphology and focal adhesion of MC3T3-E1 cells attached to the SNPs through immunostaining. The MC3T3-E1 cells grown on SNP500 were particularly well spread, with cytoplasmic extensions, compared to those grown on SNPs sintered at lower temperatures (Fig. 4c). The MTT, ALP analysis, and immunostaining results demonstrate that SNP500 with the nanopore structure similar to natural bone is most suitable for cell growth.

**Fig. 5:**
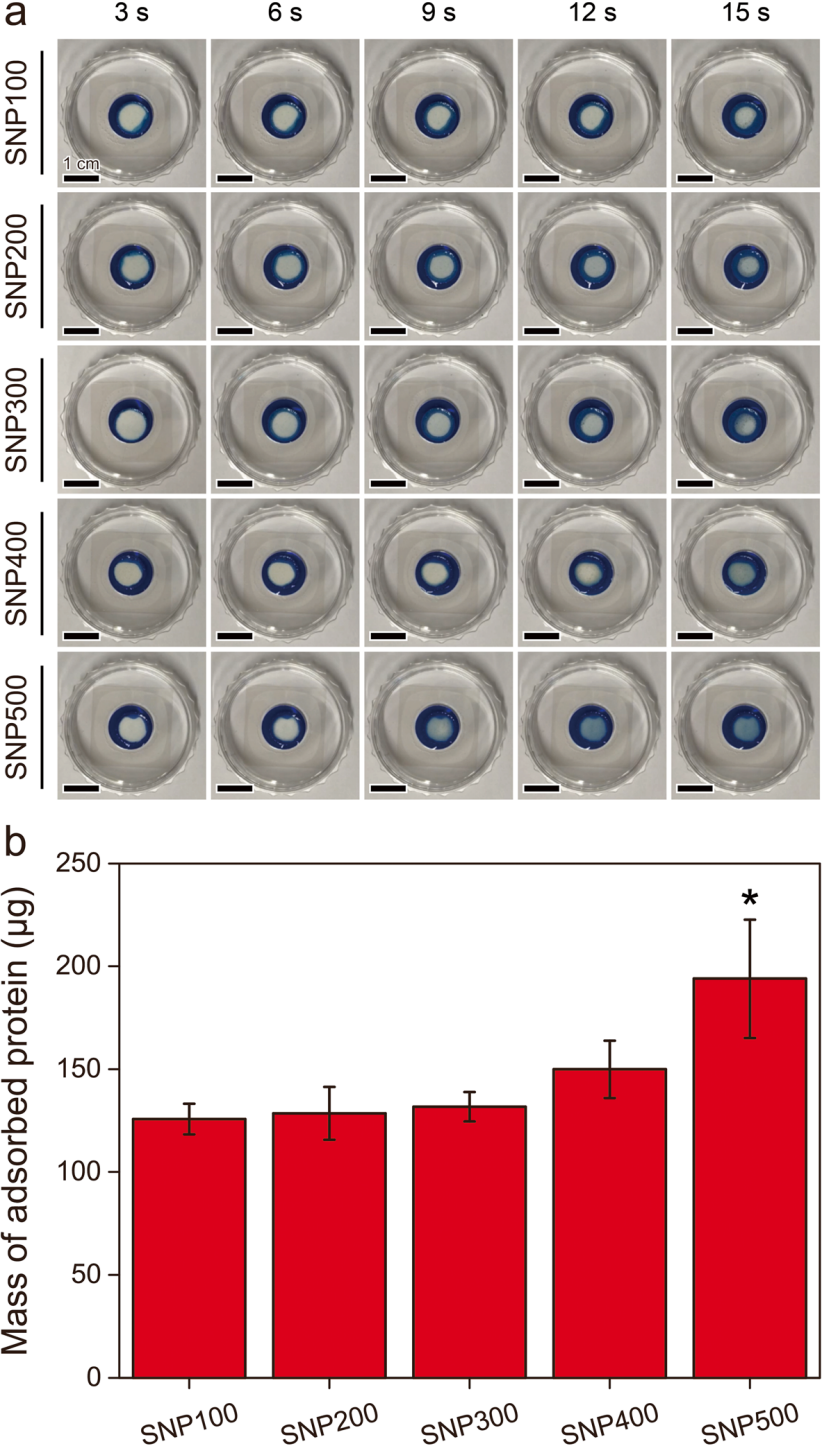
**(a)** Photographs of SNPs immersed into a methylene blue solution over time. **(b)** Protein adsorption efficiencies of SNPs prepared at different sintering temperatures (*n* = 3; *P < 0.05)

The nanoporous structures of natural bones have various important roles, including the transportation of nutrients through body fluids to promote cell growth and differentiation [78]. Hence, we compared the efficiencies of water flow and protein adsorption of scaffolds containing nanopores with different sizes. Using methylene blue as an indicator, we observed that, as the size of the nanopores increased, water flowed more quickly through the samples (Fig. 5a). This indicates that the pores inside are interconnected, and the larger porous structures are more effective in liquid flow. The amount of protein adsorbed on the samples increased with the size of the nanopores as well. Although total surface area is a very important factor that can affect the amount of protein adsorbed on the scaffold, we believe that the pore size and water flow rate are also very important factors to consider. For example, the average size of one of the most abundant proteins in the medium, bovine serum albumin (BSA), is 66.5 kDa (~ 7.1 nm) [79]. Although the size of BSA is smaller than the pore size of SNP100 (~ 16.5 nm), the effective size of BSA may be larger due to intermolecular interactions between BSA molecules and hydration in the solution [80, 81]. More importantly, when BSA molecules are adsorbed on the surface of the nanopores of SNP100, they will block the nanopores, preventing the flow of water and the movement of other BSA molecules inside the sample. We believe that this is the reason why SNP100 has the lowest mass of protein absorbed despite having the largest total surface area among the samples. On the other hand, proteins could pass through the pores of SNP 500 more easily, resulting in effective protein adsorption despite having relatively small surface area [82]. This active transportation of water and nutrients might help preosteoblasts effectively proliferate and differentiate into bone cells (Fig. 5b) [34].

### In-vivo investigation of biocompatibility and bone regeneration efficiency

**Fig. 6:**
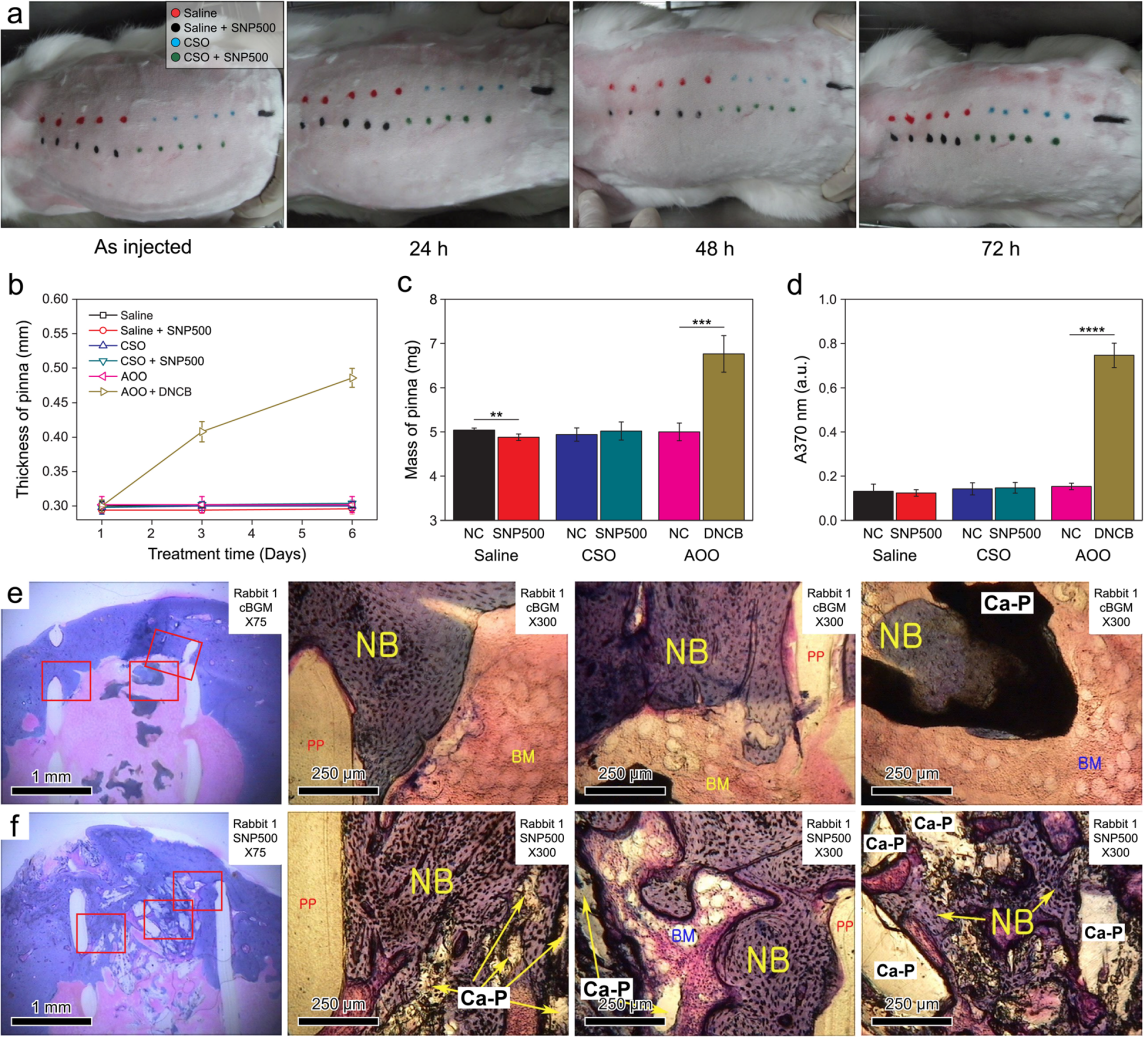
(**a**) Photographs of the back of a mouse over time following the SNP500 extract administration. **(b)** Thickness and (**c**) mass of the pinna of the mouse six days after the extract was applied on it (*n* = 5; **P < 0.01; ***P < 0.001). **(d)** Relative proliferation of lymphocytes (absorbance at 370 nm, A370 nm) after administering the extract (*n* = 5; ****P < 0.0001). There was no apparent erythema reaction or physical change when the extract was applied to the mouse, which indicates that SNP500 is highly biocompatible. Photographs of the newly formed bone tissue around (**e**) cBGM and (**f**) SNP500 12 weeks after implantation. The damaged parts were filled completely with newly formed bones around both samples. However, the average length of the bone around SNP500 was larger than that of the bone around cBGM. NB, newly formed bone; BM: bone marrow; Ca-P: implanted cBGM and SNP; PP: polypropylene

To evaluate the feasibility of using SNP as a natural bone substitute, we evaluated its biocompatibility using a mouse model. For the in-vivo test, we used SNP500 because not only the size of its nanopores (~ 30.2 nm) was similar to that of natural bones, but also it exhibited the highest proliferation and differentiation rates of preosteoblasts among the tested SNP samples [70]. SNP500 was ground into small granules (~ 1 mm). Notably, no apparent erythema or edema was observed when the extract from SNP500 was injected into the back of the mouse (Fig. 6(a) and S3). Moreover, no physical difference was observed when the SNP500 extract was applied to the pinna of the mouse. However, the thickness and mass of pinna significantly increased. The local proliferation of lymphocytes increased after DNCB treatment (Fig. 6b and c). According to the results of the local lymph node assay, the SI was only 0.95 and 1.03 when polar (saline) and nonpolar (CSO) solvents were used, respectively, whereas a high SI (4.86) was observed when DNCB was used (Fig. 6d and Table S1). These results indicate that SNP500 is highly biocompatible.

Finally, the bone regeneration efficacy of SNP500 was investigated by implantation in a rabbit model. Twelve weeks after the implantation of SNP500, the damaged parts of bones were fully filled with a newly formed bone tissue with minimal change in adjacent tissues or stimulations induced by the implanted sample (Table S2). This was similar to that observed in the control sample, cBGM. However, the average length of the newly generated bone around the implanted SNP500 (0.985 mm) was larger than that around the cBGM (0.897 mm) (Fig. 6e, f, Fig. S4, and Table S3). In this regard, natural bone mimicking SNP500, prepared using a low-temperature sintering process considerably promotes better bone regeneration than cBGM. This result is noteworthy as cBGM is a two-phase material mixed with 20% HA and 80% beta-TCP, while SNP500 is only composed of HA [83, 84]. Therefore, we believe that the nanopores have an essential role in increasing the bone regeneration efficiency of BGMs. Furthermore, natural bone-mimicking nanopores may be one of the key factors to consider in developing highly efficient next-generation alloplastic BGMs.

## Discussion

Typically, nanopores of natural bones cause an asymmetrical division of stem cells into osteoblasts [28, 37]. Moreover, the geometry of HAP, such as nanopores, is a critical parameter affecting bone induction [30, 85]. However, it has been very challenging to mimic nanopores into alloplastic materials, including HAP. At general sintering temperatures beyond 1000 °C, coalescence and destruction of pores occur in scaffolds. Among them, nanopores mostly disappear, compared to other pores such as macro- and micro-pores [58, 59].

In this report, we propose a simple yet powerful method to introduce nanopores, which exist in natural bones, into alloplastic HAP BGMs by simply pressing HAP NPs and sintering the pellet within a low-temperature range. We hypothesized that low-temperature sintering plays an important role in the introduction of nanopores into the HAP-based scaffold. In the low-temperature range of sintering, coalescence of vacancies between HAPs occurs, resulting in formation of nanopores in the HAP-based scaffold (Fig. S5). Since the coalescence of vacancies between these HAPs is very sensitive, nanopores of different sizes can be finely produced by controlling the sintering temperature to the low temperature ranges.

To verify our hypothesis, we synthesized HAP NPs and then compressed them. The prepared HAP pellets were sintered within a low-temperature range. We controlled the sintering temperature from 100 to 500 °C to fabricate nanopores with different sizes. Consequently, we introduced nanopores into the HAP-based scaffold with sizes between 16.5 and 30.2 nm. Notably, nanopores with a size of ~ 30.2 nm, similar to those of natural bone, were obtained. The introduction of nanopores in HAP-based scaffolds helps promote cell proliferation and differentiation rates. In addition, these nanopores improve the water flow and protein adsorption in HAP-based scaffolds. Furthermore, we investigated the bone regeneration efficacy of the HAP-based scaffold containing nanopores (SNP500) by implanting it in a rabbit model. The length of the newly generated bone around the implanted SNP500 was larger than that around the cBGMs (0701 M + G01, Biomatlante) containing micro- and macropores. This study is valuable because it provides a straightforward and powerful method to introduce finely controlled nanopores into artificial scaffolds and reveals the importance of nanopores in bone regeneration.

## Conclusions

We report a simple yet powerful method to introduce nanopores, which exist in natural bones, into alloplastic HAP BGMs by simply pressing HAP NPs and sintering the pellet within a low-temperature range. The size of the nanopores could be finely controlled by changing the sintering temperature. HAP SNPs with sizes of ~ 30.2 nm, similar to those of pores in natural bones, promoted cell proliferation and differentiation rates. The HAP SNPs not only were highly biocompatible, but also could promote bone regeneration more effectively than the commercially available bone graft material when were implanted into small animals. Based on these results, we believe that nanopores may be a crucial factor to consider in fabrication of highly effective next-generation alloplastic BGMs.

## Supplementary Information

**Additional file 1.**

## Data Availability

All data generated or analyzed during this study are included in this published article.

## Abbreviations

ALP: alkaline phosphate
AOO: acetone olive oil
BGM: bone graft materials
CSO: cotton seed oil
FBS: fetal bovine serum
PBS: phosphate-buffered saline
SEM: scanning electron microscopy
SI: stimulation index
TCP: tricalcium phosphate
TEM: transmission electron microscopy
XRD: X-ray diffraction
