# Supplementary material for: Natural bone-mimicking nanopore-incorporated hydroxyapatite scaffolds for enhanced bone tissue regeneration
Source: Biomater Res. 2022 Feb 25;26:7. doi: 10.1186/s40824-022-00253-x (PMC8876184; doi:10.1186/s40824-022-00253-x)
Supplement: Supplementary file 1 — Additional file 1. [file 40824_2022_253_MOESM1_ESM.docx]

Supporting Information

**Natural bone-mimicking nanopore-incorporated hydroxyapatite scaffolds for enhanced bone tissue regeneration**

*Chansong Kim^1‡^, Jin Woong Lee^1,2‡^, Jun Hyuk Heo^1,2,*^, Cheolhyun Park^1^, Dai-Hwan Kim^1^, Gyu Sung Yi^1^, Ho Chang Kang^3^, Hyun Suk Jung^1^, Hyunjung Shin^4^, and Jung Heon Lee^1,2,5,6,*^*

^1^School of Advanced Materials Science and Engineering, Sungkyunkwan University, Suwon 16419, Republic of Korea

^2^Research Center for Advanced Materials Technology, Sungkyunkwan University, Suwon 16419, Republic of Korea

^3^Probiomimetic Research Institute, Bundang Technopark, Seongnam 13219, Republic of Korea

^4^Department of Energy Science, Sungkyunkwan University, Suwon 16419, Republic of Korea

^5^Biomedical Institute for Convergence at Sungkyunkwan University, Sungkyunkwan University, Suwon 16419, Republic of Korea

^6^Institute of Quantum Biophysics (IQB), Sungkyunkwan University, Suwon 16419, Republic of Korea

*Corresponding authors

E-mail: saegusa@skku.edu (Heo, J. H.); jhlee7@skku.edu (Lee, J. H.)

‡These authors contributed equally to this study.


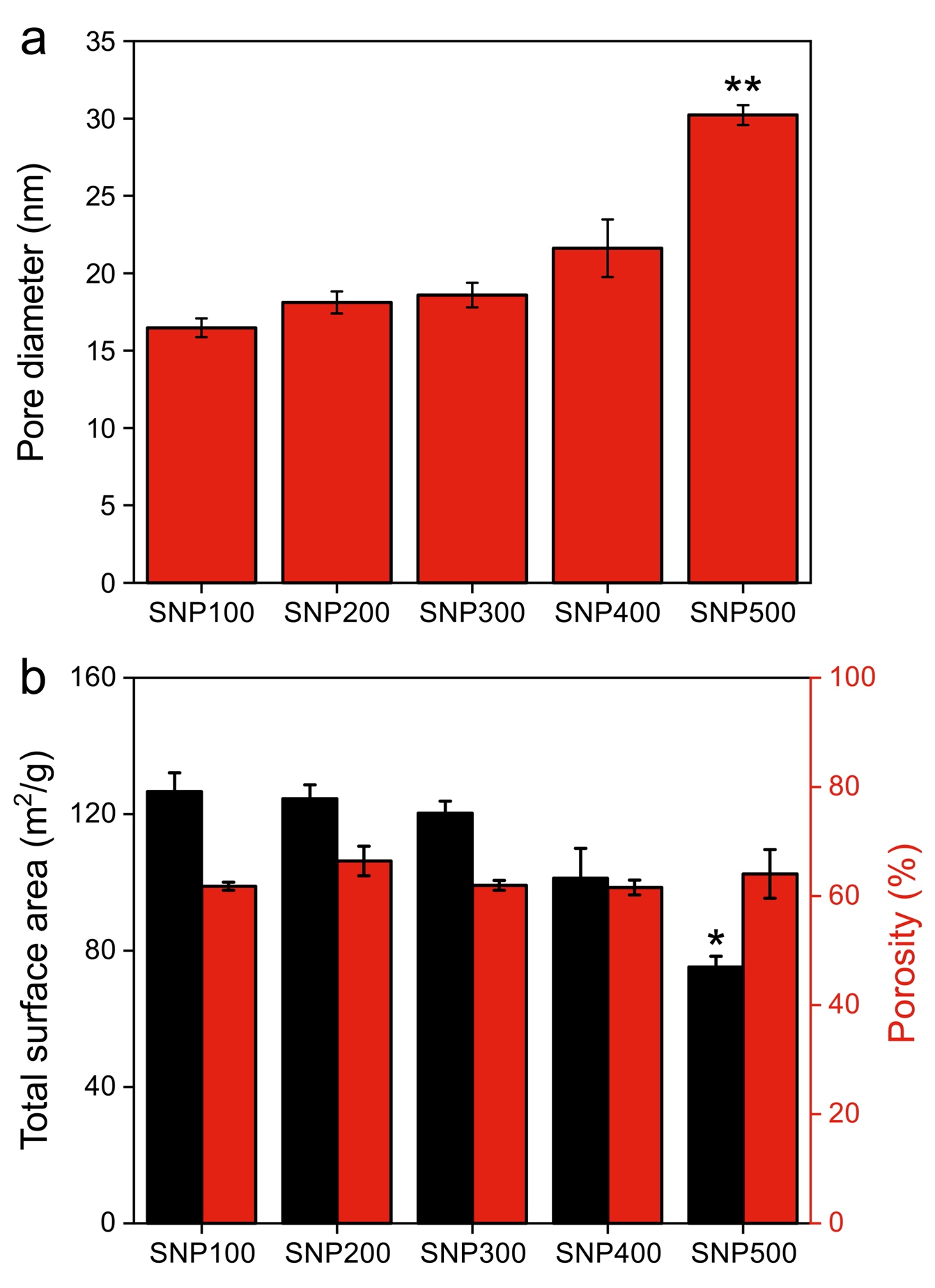


Fig. S1. (a) Pore diameters, (b) total surface areas, and porosities of SNPs sintered at different temperatures. The statistical significance was derived relative to the results of SNP100 (n = 3; *p < 0.05; **p < 0.01).


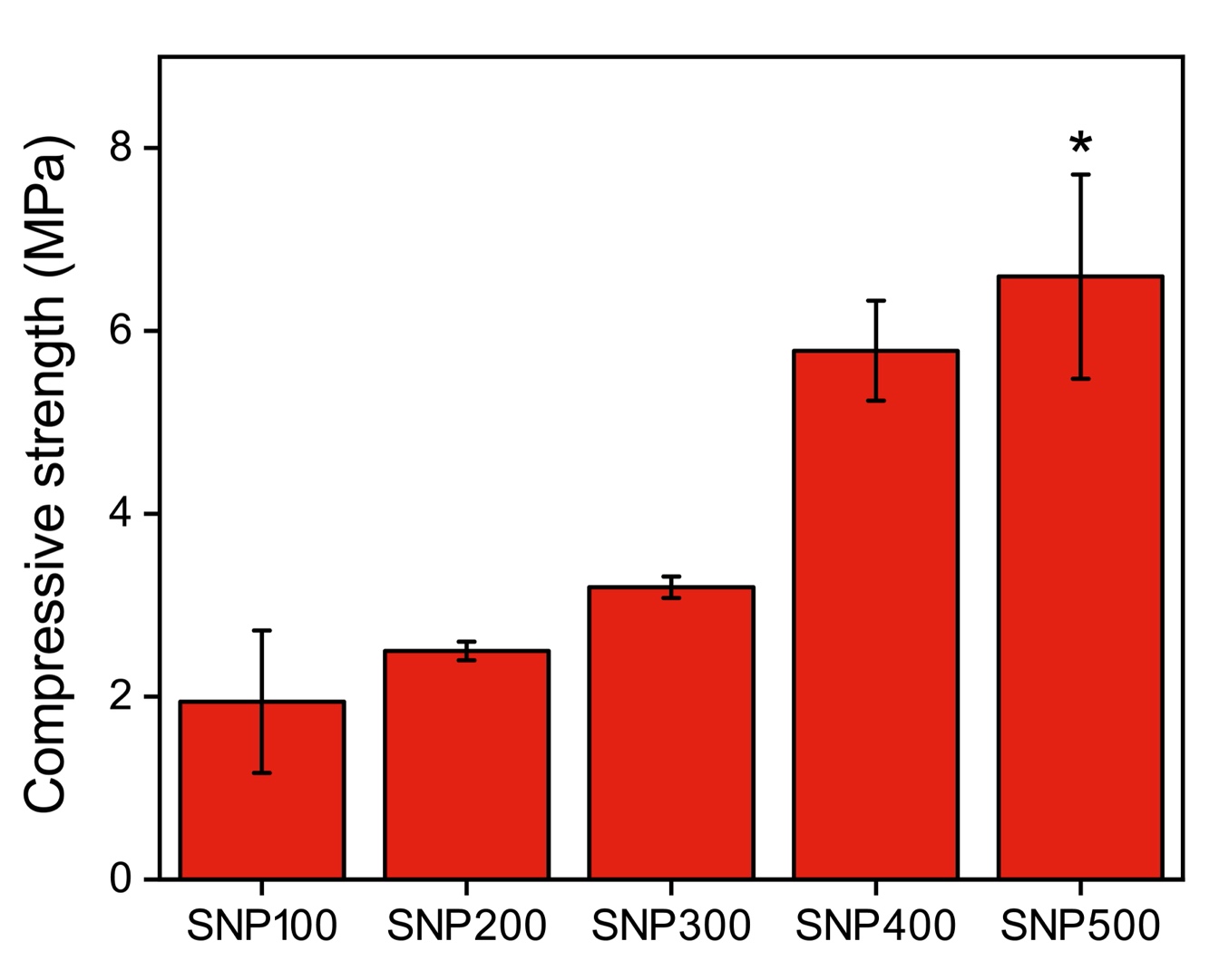


Fig. S2. Compressive strength of SNPs sintered at different temperatures. The statistical significance was derived relative to the results of SNP100 (n = 3; *p < 0.05).


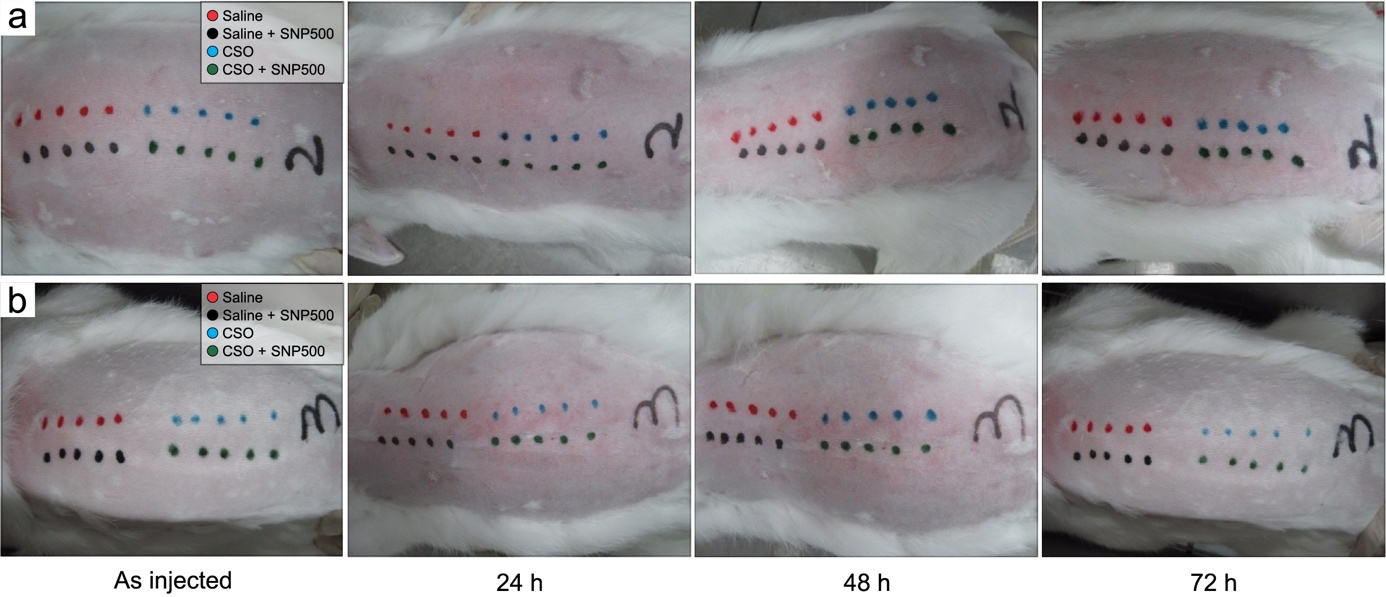


Fig. S3. Photographs of the back of (a) mice 2 and (b) 3 over time following the administration of SNP500 extract. No apparent erythema reaction was observed when the extract was injected into the mouse, indicating that SNP500 is highly biocompatible. NC, negative control; CSO, cotton seed oil.


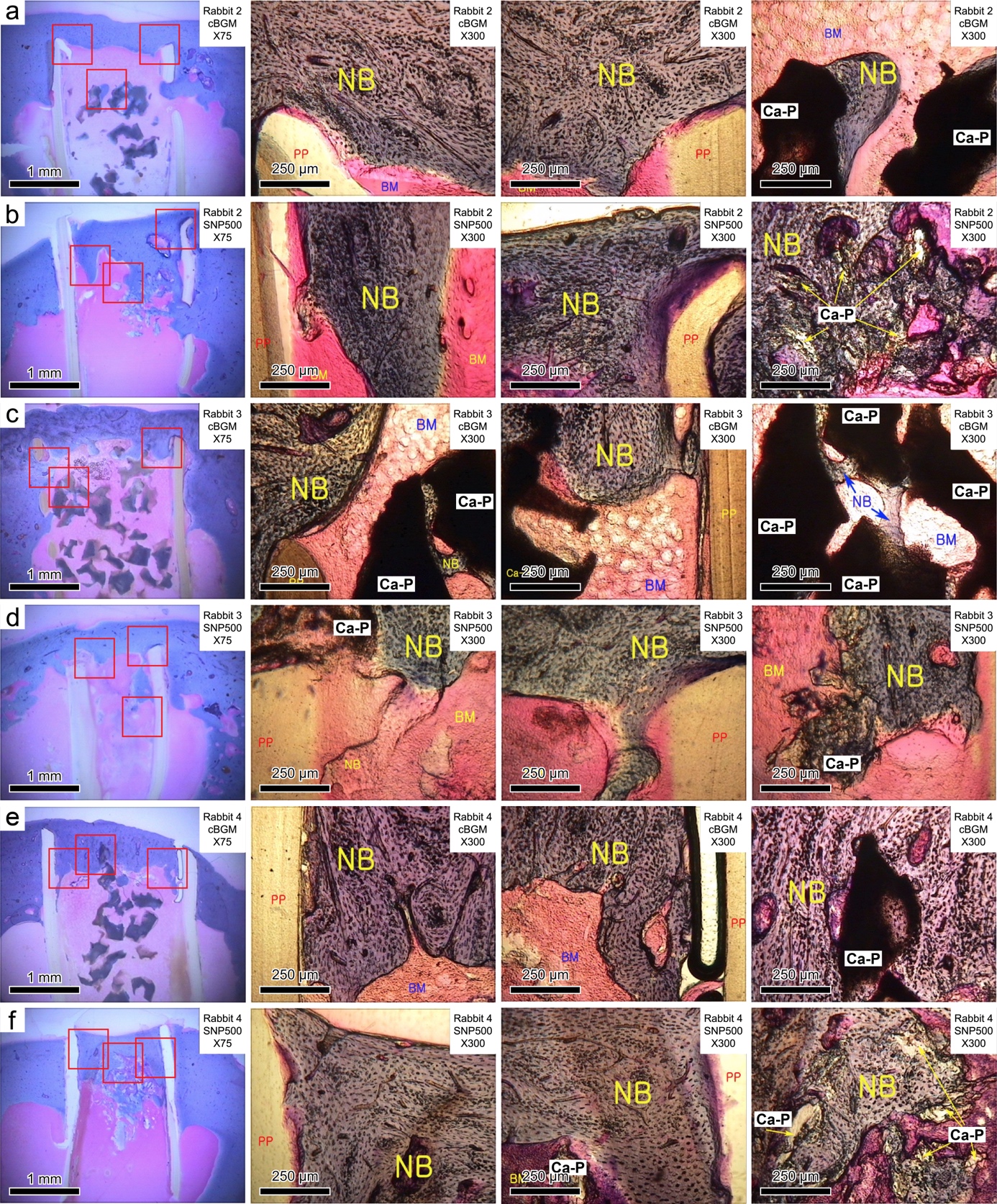


Fig. S4. Photographs of newly formed bones around (a) cBGM and (b) SNP500 after 12 weeks of implantation in rabbits 2 and 3 ([c] cBGMs and [d] SNP500s) and 4 ([e] cBGMs and [f] SNP500s). NB, newly formed bone; BM: bone marrow; Ca-P: implanted cBGM and SNP500; PP: polypropylene.

**
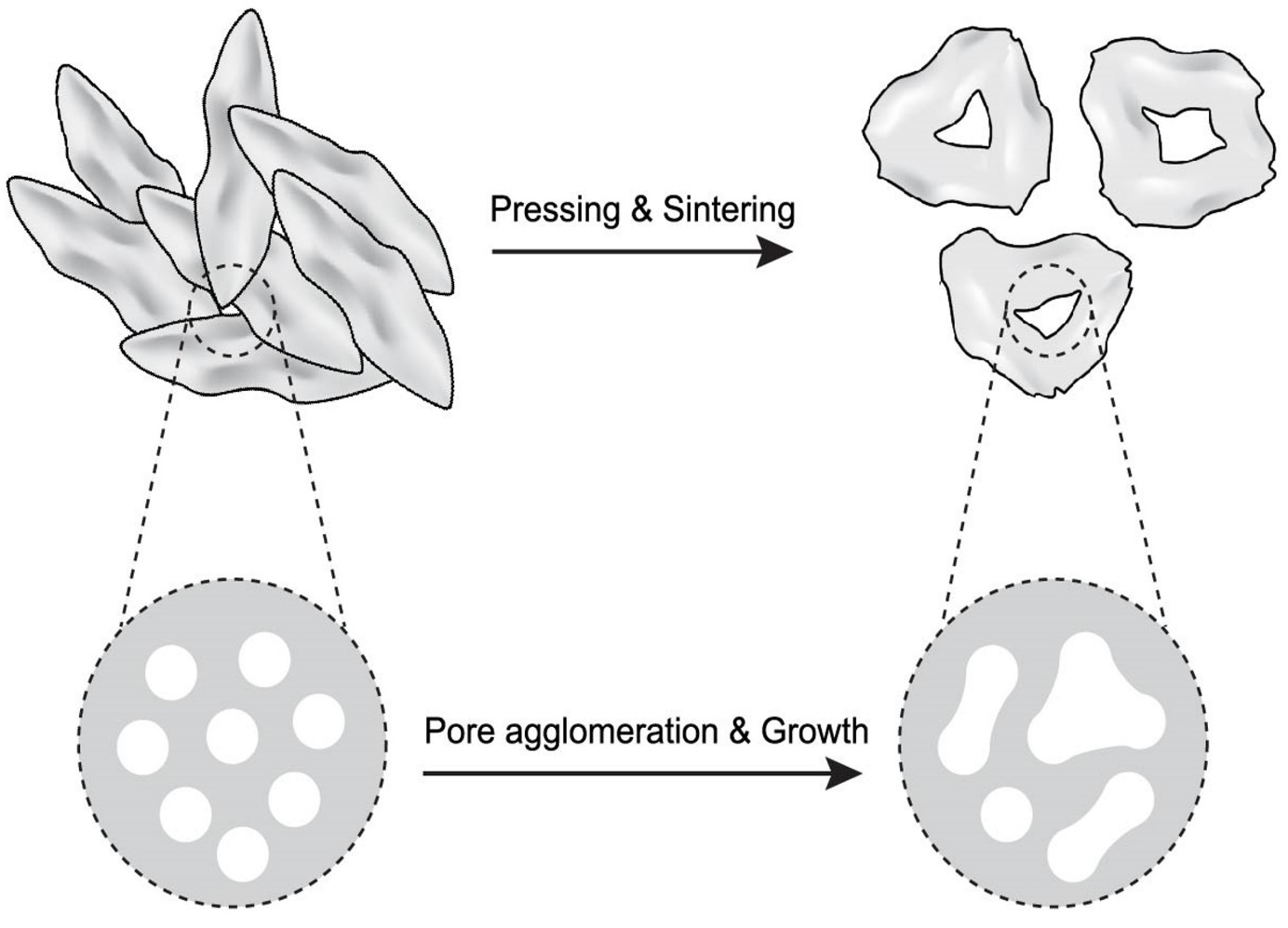
**

Fig. S5. Schematic possible mechanism of nano-pore formation with SNP via low sintering temperature.

**Table S1.** Apparent intensity of erythema reaction when the extracts were applied to the pinna of the mouse. NC, negative control; CSO, cotton seed oil; AOO, acetone and olive oil.

|  |  | NC | | | | |  | Experimental | | | | |
| --- | --- | --- | --- | --- | --- | --- | --- | --- | --- | --- | --- | --- |
|  |  | NC1 | NC2 | NC3 | NC4 | NC5 |  | E1 | E2 | E3 | E4 | E5 |
| Saline | D+1 | 0 | 0 | 0 | 0 | 0 |  | 0 | 0 | 0 | 0 | 0 |
|  | D+3 | 0 | 0 | 0 | 0 | 0 |  | 0 | 0 | 0 | 0 | 0 |
|  | D+6 | 0 | 1 | 0 | 1 | 0 |  | 1 | 0 | 1 | 0 | 0 |
|  |  |  |  |  |  |  |  |  |  |  |  |  |
| CSO | D+1 | 0 | 0 | 0 | 0 | 0 |  | 0 | 0 | 0 | 0 | 0 |
|  | D+3 | 0 | 0 | 0 | 0 | 0 |  | 0 | 0 | 0 | 0 | 0 |
|  | D+6 | 1 | 0 | 1 | 1 | 0 |  | 0 | 1 | 1 | 0 | 0 |
|  |  |  |  |  |  |  |  |  |  |  |  |  |
| AOO | D+1 | 0 | 0 | 0 | 0 | 0 |  | 0 | 0 | 0 | 0 | 0 |
|  | D+3 | 0 | 0 | 0 | 0 | 0 |  | 3 | 3 | 3 | 3 | 3 |
|  | D+6 | 0 | 0 | 1 | 1 | 0 |  | 4 | 4 | 4 | 4 | 4 |

1, none; 2, barely perceptible; 3, well-defined, 4, moderate; 5, severe

Table S2. Tissue response to cBGMs and SNP500s implantations in the rabbits. (No immune reaction occurred when cBGMs and SNP500s were implanted, indicating that both samples showed biocompatibility).

|  | cBGM | | | |  | SNP500 | | | | |
| --- | --- | --- | --- | --- | --- | --- | --- | --- | --- | --- |
|  | **Test1** | **Test2** | **Test3** | **Test4** |  | **Test1** | **Test2** | **Test3** | **Test4** |  |
| Stimulation | None | None | None | None |  | None | None | None | None |  |
| Poly-morphonuclear  leukocyte | None | None | None | None |  | None | None | None | None |  |
| Lymphocyte | None | None | None | None |  | None | None | None | Rare |  |
| Plasma cell | None | None | None | None |  | None | None | None | None |  |
| Macrophage | None | None | None | None |  | None | None | None | None |  |
| Giant cell | None | None | None | None |  | None | None | None | None |  |
| Parenchyma necrosis | None | None | None | None |  | None | None | None | None |  |
| Pathological angiogenesis | None | None | None | None |  | None | None | None | None |  |
| Fibrosis | None | None | None | None |  | None | None | None | None |  |
| Fatty  infiltration | None | None | None | None |  | None | None | None | None |  |
| Traumatic necrosis | Normal | Normal | Normal | Normal |  | Normal | Normal | Normal | Normal |  |
| Fragment of material | None | None | None | None |  | None | None | None | None |  |
| Untreated tissue | Normal | Normal | Normal | Normal |  | Normal | Normal | Normal | Normal |  |

Table S3. The average length of the newly generated bone around the implanted SNP500 and cBGM (n = 4 for each sample).

|  | cBGM |  | SNP500 | |
| --- | --- | --- | --- | --- |
| Length of the new bone | 0.897 $\pm$ 0.280 |  | 0.985 $\pm$ 0.480 |  |
| *p* value | 0.590 | | |  |
